# Effects of Indocyanine green on cultured retinal ganglion cells in-vitro

**DOI:** 10.1186/1756-0500-2-236

**Published:** 2009-11-25

**Authors:** S Balaiya, Vikram S Brar, Ravi K Murthy, KV Chalam

**Affiliations:** 1 University of Florida College of Medicine, Department of Ophthalmology Jacksonville, FL, USA

## Abstract

**Background:**

Indocyanine green (ICG) dye is commonly used to stain the inner limiting membrane during macular surgery. There are reports documenting the toxicity of ICG on retinal pigment epithelial cells, with conflicting results in retinal ganglion cells. In the present study, we evaluated the effect of ICG on retinal ganglion cells in vitro.

Cultured rat retinal ganglion cells (RGC-5) were exposed to different concentrations of ICG (0.25, 0.5, 1.0, 1.25, & 5 mg/ml) and at various time intervals (1, 5, 15, 30, & 60 minutes). Changes in structural morphology were identified using phase contrast bright field microscopy. Cell viability was quantified using the neutral red assay and cell death was characterized using Annexin-V staining.

**Findings:**

Significant morphologic changes were observed at the 15 and 60 min intervals for all concentrations, where a reduction in cell size and loss of normal spindle shape was noted. A dose dependent decrease in cell viability was observed with increasing concentration of ICG as well as increasing exposure intervals. Compared to control, 48-74% reduction in neutral red uptake at all concentrations for exposures 5 min or greater (p < 0.001). Even at 1 min exposure, a dose dependent decline was observed in cell viability, with a 28-48% decline for doses above 1.25 mg/ml (p = 0.007). Staining with Annexin-V, demonstrated a similar dose and time dependent increase in number of cells exhibiting early apoptosis. A greater than two-fold increase in Annexin-V expression for all doses at exposures greater than 1 min was noted.

**Conclusion:**

ICG dye exhibits toxicity to retinal ganglion cells at clinically relevant doses following 1 min exposure.

## Background

Indocyanine green (ICG) is commonly used to stain the internal limiting membrane (ILM) [1,2] during macular surgery for the treatment of idiopathic macular holes [3-5] and diffuse diabetic macular edema [6]. However, the safety of intravitreal use of ICG is not well established. Adverse effects such as visual field defects [7-9] and atrophy of the retinal ganglion cell layer [10] subsequent to ICG assisted membrane peeling have been reported. The underlying cause of ICG-related adverse effects has been proposed to be due to the osmolarity of the solution [11] or photochemical damage [12].

The retinal ganglion cell (RGC) layer is the first to come in contact with ICG dye used for staining in macular surgery and theoretically has the maximum exposure to the dye. A number of animal and in-vitro studies have evaluated the toxicity of ICG in cell culture models [13-18]. However, there is conflicting data with regards to presence of ICG mediated toxicity.

In this study, we investigated the effect of different ICG concentrations at specific time intervals on rat RGCs (RGC-5), in vitro, to establish a safe dose for use in-vivo. We specifically evaluated the effect of ICG on morphology, cell viability, and mechanism of cell death.

## Methods

### Cell Culture

Rat retinal ganglion cells (RGC-5) were graciously donated by Dr. Neeraj Agarwal, University of North Texas. RGC-5 cells were maintained in log-rhythmic growth and cultured in Dulbecco's modified Eagle's medium (DMEM: L-glutamine, 110 mg/L sodium bicarbonate and 1 g/L D-glucose) containing 10% fetal bovine serum (JRH Biosciences, Lenexa, Ka) and 100 U/ml of penicillin and 100 μg/ml of streptomycin. The cells were maintained in 75 cm^2 ^filter-capped cell culture flasks and incubated with 5% CO_2 _at 37°C.

### ICG Preparation

25 mg of ICG (Acorn, IL) was diluted in 5 ml phosphate-buffered saline with albumin (PBSA) to obtain a 5 mg/ml mixture. The prepared solution was further diluted to obtain ICG concentrations of 0.25, 0.5, 1.0, 1.25, and 5 mg/ml. ICG solution was prepared fresh for each experiment. The osmolarity of the prepared ICG solutions ranged between 309 and 313 mosm/litre.

### ICG Staining

The RGCs were grown in 24-well culture plates (Corning, Corning, NY) for 24 hours in conditioned cell culture media. The culture medium was aspirated from each of the 24-well culture plates and replaced with the various ICG concentrations. RGCs were exposed to the various concentrations of ICG (0, 0.25, 0.5, 1.0, 1.25 and 5 mg/ml) for 1, 5, 15, 30 and 60 minutes. Cultures containing PBSA alone served as control. Following exposure to ICG dye, cells were washed with PBS and were subsequently cultured for 2 hours in DMEM, at which point the experiments were concluded.

### Structural Morphology

Glass cover slips with RGC cells exposed to different ICG concentrations (0, 0.25, 0.5, 1.0, 1.25, 5 mg/ml) were washed twice with PBS and mounted on a slide using crystal mount solution. The slides were analyzed by bright field microscopy (Olympus U-RFL-T) to identify morphological changes.

### Cell viability by Neutral Red (NR) Uptake Assay

The NR uptake assay was done as previously described [19]. Briefly, NR working solution (0.033%) was freshly prepared for each experiment by diluting 1 ml of NR stock solution (0.5% (Sigma Aldrich, St. Louis, MO) in 14.5 ml of DEPC (Sigma Aldrich, St. Louis, MO). After ICG exposure, an equal volume of fresh media supplemented the ICG solution containing 33 μl of NR working solution. The cells were allowed to incubate at room temperature (RT) for 2 h. After incubation, the NR solution was aspirated and the attached cells were washed twice with PBS, before allowing too air-dry at RT for 20 minutes. The cells were treated with ice-cold solubilization buffer (1% acetic acid/50% ethanol; 300 μL) to release the remaining NR from the internal compartments of the cell for 30 minutes. The absorbance was measured at 490 nm using a Bio-Tek ELX 800 microplate reader.

### Evaluation of Apoptosis with Flow Cytometry

8 × 10^3 ^RGC-5 cells were isolated and centrifuged. Cells were then washed twice with PBS and resuspended in 1× binding buffer (10 mM HEPES/NaOH, 140 mM NaCl, and 2.5 mM CaCl_2_, pH 7.5). 500 μl of each experimental cell suspension was added to different tubes and stained with 5 μl of Annexin V-FITC. After incubating the tubes at room temperature for 10 minutes, the fluorescence was analyzed by flow cytometry (Epics XL-MLC, Becton Dickinson, Mountain View, CA). The 0.25, 0.50, and 1.0 mg/ml concentrations of ICG were evaluated.

### Statistical analysis

Statistical analysis of the data was done using Student's *t *test (Graphpad Prizm, Version3, CA, US). A p value of less than 0.05 was considered significant.

## Results

### Cell Morphology

**Figure 1:**
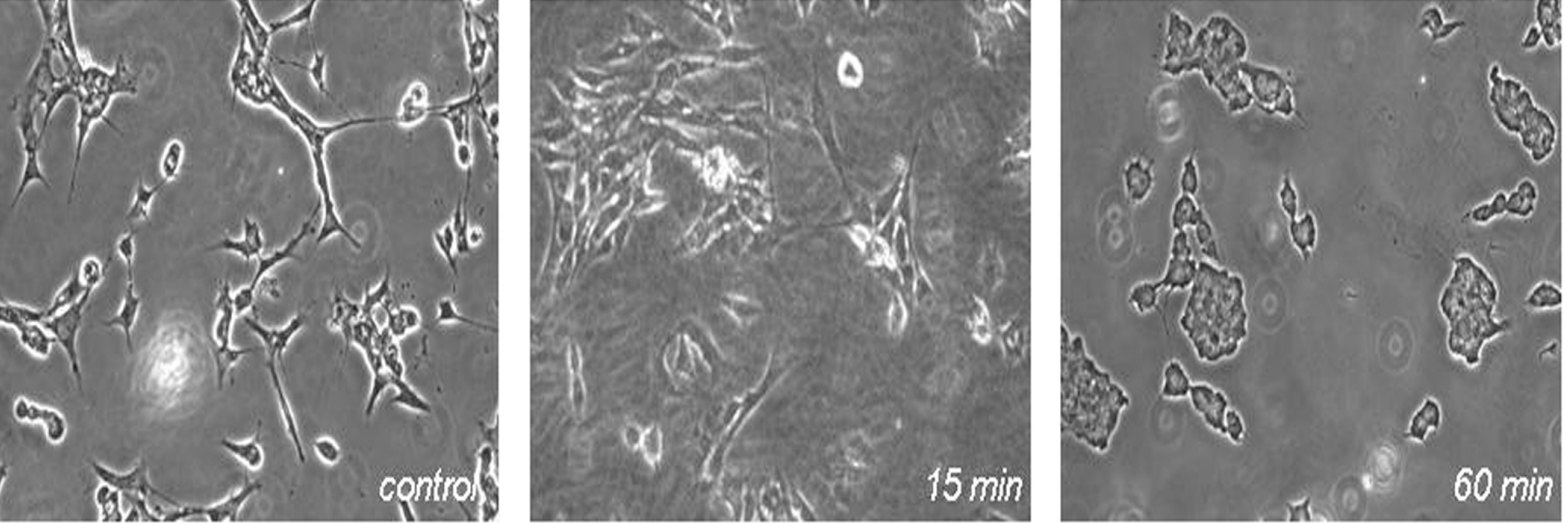
**Morphologic changes following exposure to ICG dye**. Representative images at 0.5 mg/ml concentration were obtained with phase contrast microscopy. Cells became rounder and smaller with increasing duration of exposure.

After 1 and 5-minute incubation at all ICG concentrations, the cells continued to show similar structural features compared to controls. However, there was a significant morphological variation after 15 and 60-minute incubation, at all concentrations. The cells assumed an oval shape and were reduced in size compared with longer spindle shaped control. (Figure 1)

### Cell Viability Assay (Neutral Red)

RGC cells exposed to concentrations as low as 0.25 & 0.5 mg/ml demonstrated reduced NR uptake as early as 1 min. A 12 and 14% reduction in cell viability was noted respectively. At the same exposure time, the 1.0 & 1.25 mg/ml doses, demonstrated a 21 and 28% reduction, with a 43% reduction noted at the highest concentration (p = 0.007).

**Figure 2:**
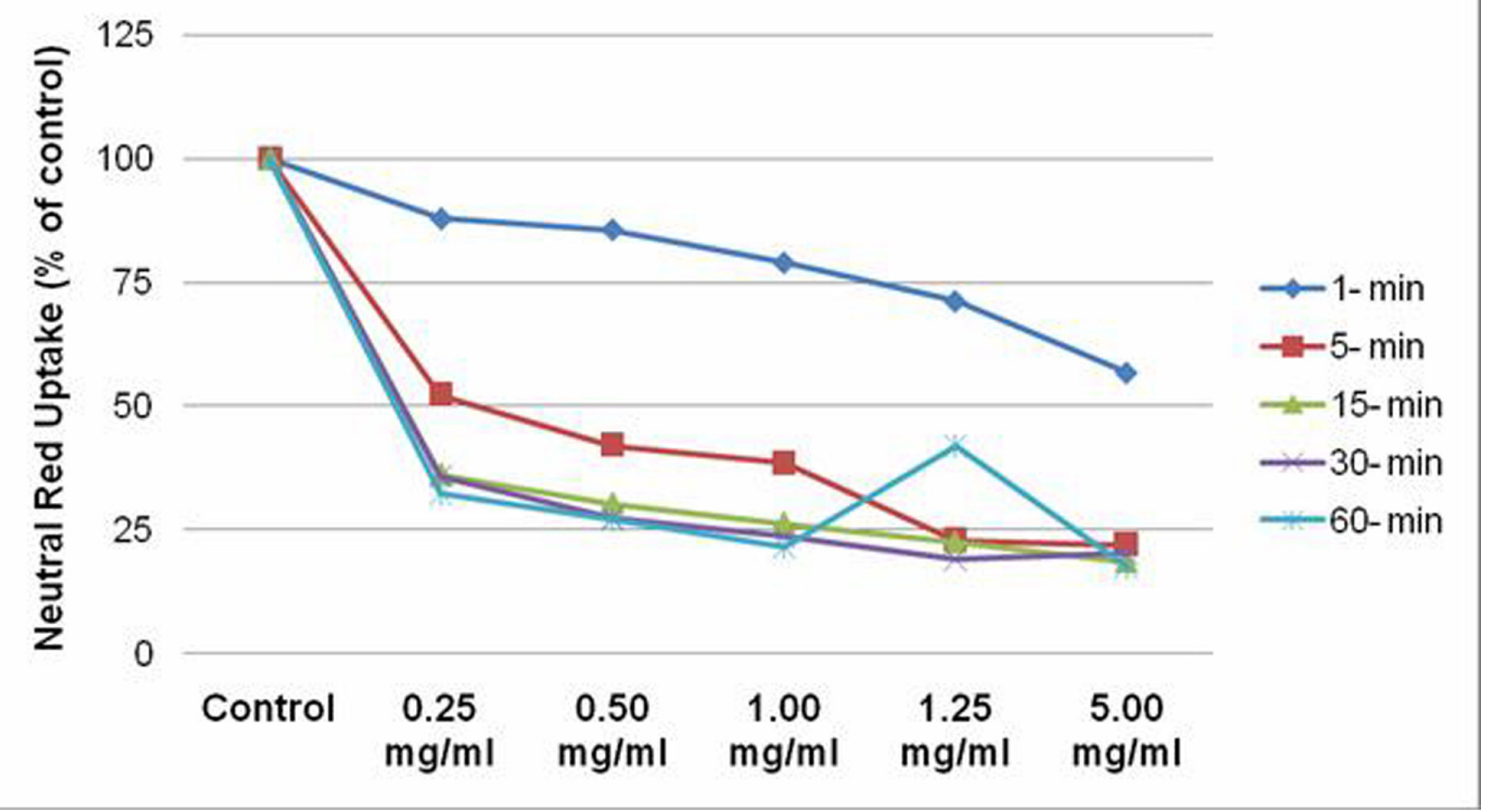
**Cell viability evaluated by Neutral Red assay, demonstrates a dose and time dependent decrease in cell viability following ICG exposure**. Data expressed as percent of control.

After 5 min exposure, the lowest concentration group, reduced cell viability by 48% compared with control. This trend continued with either an increase in dose or exposure time. The greatest reduction in cell viability was observed at the highest concentration at the longest exposure, where only 17.5% of the remaining cells were viable (p < 0.001) (Figure 2).

### Flow Cytometry

Flow cytometric analysis, revealed a parallel increase in Annexin-V detection compared with the results of the neutral red assay. Following 1 min exposure, both 0.25 and 0.50 mg/ml concentrations, demonstrated a 45% increase in apoptotic cells compared with control. Similar to the results from the cell viability assay, increased duration at the lowest concentration, correlated with a 2.5 fold increase in percentage of apoptotic cells.

**Figure 3:**
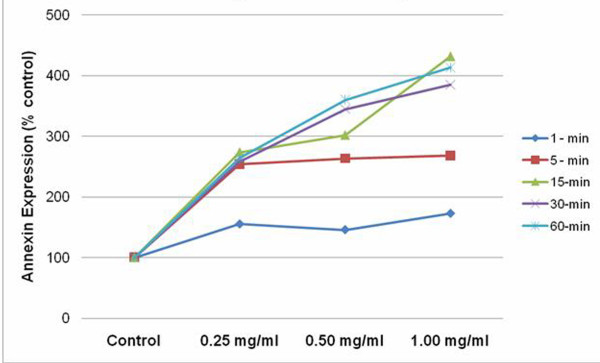
**Annexin-V detection evaluated by flow cytometry, demonstrates a dose and time dependent increase in early apoptosis following ICG exposure**. Data expressed as percent of control.

The greatest separation among the various doses was evident at 15 min exposure interval. The 0.25 mg/ml concentration maintained a 2.5 fold increase in apoptotic cells, where a 3 and 4 fold increase occurred for the 0.5 and 1.0 mg/ml concentrations respectively. A similar relationship was maintained across the various doses for the remaining exposure times. (Figure 3)

## Discussion

Indocyanine green (ICG) is an amphillic tricarbocyanine dye often used in macular surgery, where it stains the internal limiting membrane and facilitates its removal [1-6]. Both clinical [7-10] and experimental [11-17] reports have documented toxicity of ICG dye. However, there is a lack of standardization with regards to doses tested and exposure times evaluated. This study addresses this question by testing different concentrations (including the concentrations used in clinical setting: 0.25 and 0.5 mg/ml) at different exposure times, while adding to the body of literature relating to the mechanism of ICG mediated cell death.

Morphologic analysis demonstrated the toxic effects of ICG on retinal ganglion cells where evidence of toxicity was observed with increased exposure time. This becomes clinically relevant in cases of incomplete ICG removal or post surgical persistence of the dye [20]. These results were further supported by loss of cell viability in a dose and time dependent manner, where ICG toxicity was observed at the lowest concentration after 1 min exposure. It has been suggested that 1.25 mg/ml, provides the optimal visibility of the ILM in clinical settings [21]. Our study demonstrated 29% reduction in cell viability at this concentration, following 1 min exposure. This is corroborated by clinical studies which have reported visual filed defects after in-vivo use of 0.05% ICG for 1 minute during vitrectomy for macular hole surgery [7,9].

Mechanistic studies have revealed that exposure to ICG results in apoptosis in the retinal cells [17]. In our study, we used Annexin-V, a human anticoagulant that has strong attraction for phoshatidylserine residues, to detect apoptosis in the RGC cell line. Our results indicated as both time and concentration increased, the detection of Annexin-V increased. For doses = 0.50 mg/ml, 1 minute exposure resulted in approximately 50% increase in apoptosis. However, at exposure time of 5 minutes, significant cell loss (>2-fold) was observed irrespective of the concentration of the dye used. These results mirrored the loss in cell viability using the neutral red (NR) assay.

The limitation of the study is the use of an in-vitro model which does not factor the penetrability of ICG across the ILM.

## Conclusion

Our work illustrates worsening cytotoxic effects of ICG to retinal ganglion cells in a dose and time-dependent manner. In addition, we have shown that cytotoxic effect occurs in the absence of any stimulating light. We suggest that the lower concentration and shorter staining time of ICG be used for staining ILM during vitrectomy. Complete and quick removal after injection over the macula is imperative to prevent detrimental changes to the retinal ganglion cells.

## List of abbreviations

ICG: Indocyanine green; RGC: Retinal ganglion cell; NR: Neutral red.

## Competing interests

The authors declare that they have no competing interests.

## Authors' contributions

KVC was involved in the conception and design of the study; SB was involved in the acquisition of data; VSB and RK were involved in the analysis of the data and preparation of the manuscript.

All authors have read and approved the final manuscript.
